# Supplementary material for: Measuring the reproducibility and quality of Hi-C data
Source: Genome Biol. 2019 Mar 19;20:57. doi: 10.1186/s13059-019-1658-7 (PMC6423771; doi:10.1186/s13059-019-1658-7)
Supplement: Supplementary file 1 — Supplementary figures and tables describing additional results and the datasets used in this study, respectively. (DOCX 5 kb) [file 13059_2019_1658_MOESM1_ESM.docx]

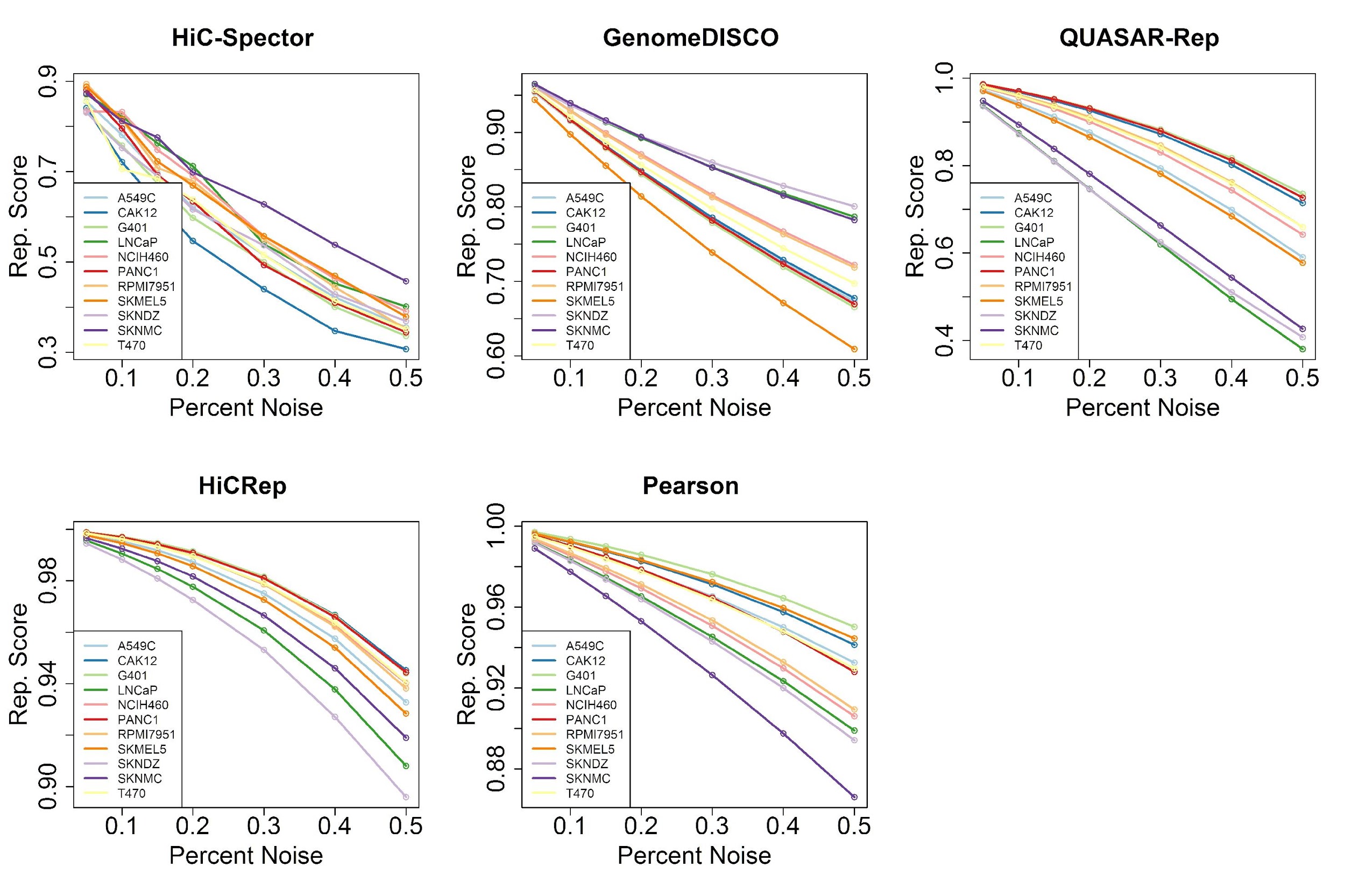
Figure S1. Reproducibility scores assigned to each noise injected replicate pair for each cell type for 33% random ligation configuration. For every cell type and every measure, we see a monotonic trend of decreasing scores with higher levels of noise. The same trends for each measure are observed in the 66% random ligation noise configuration (not shown).


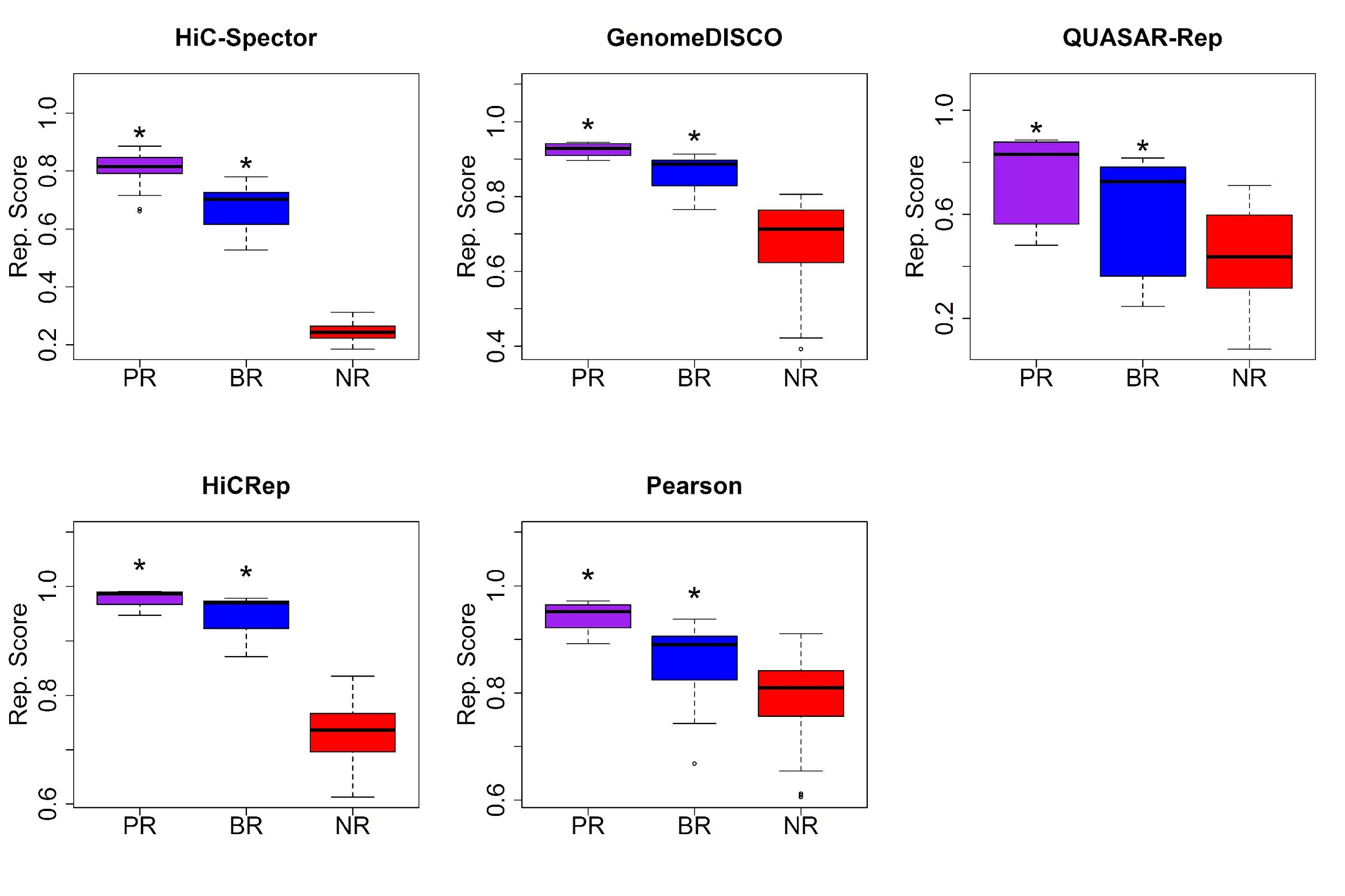


Figure S2. Boxplots showing the distribution of reproducibility scores assigned to each replicate pair category by each measure: pseudo replicates (PR), biological replicates (BR) and non-replicates (NR). Asterisks indicate that the marked distribution is significantly larger than the preceding one according to a one-sided Kolmogorov-Smirnov test (P < 0.01).


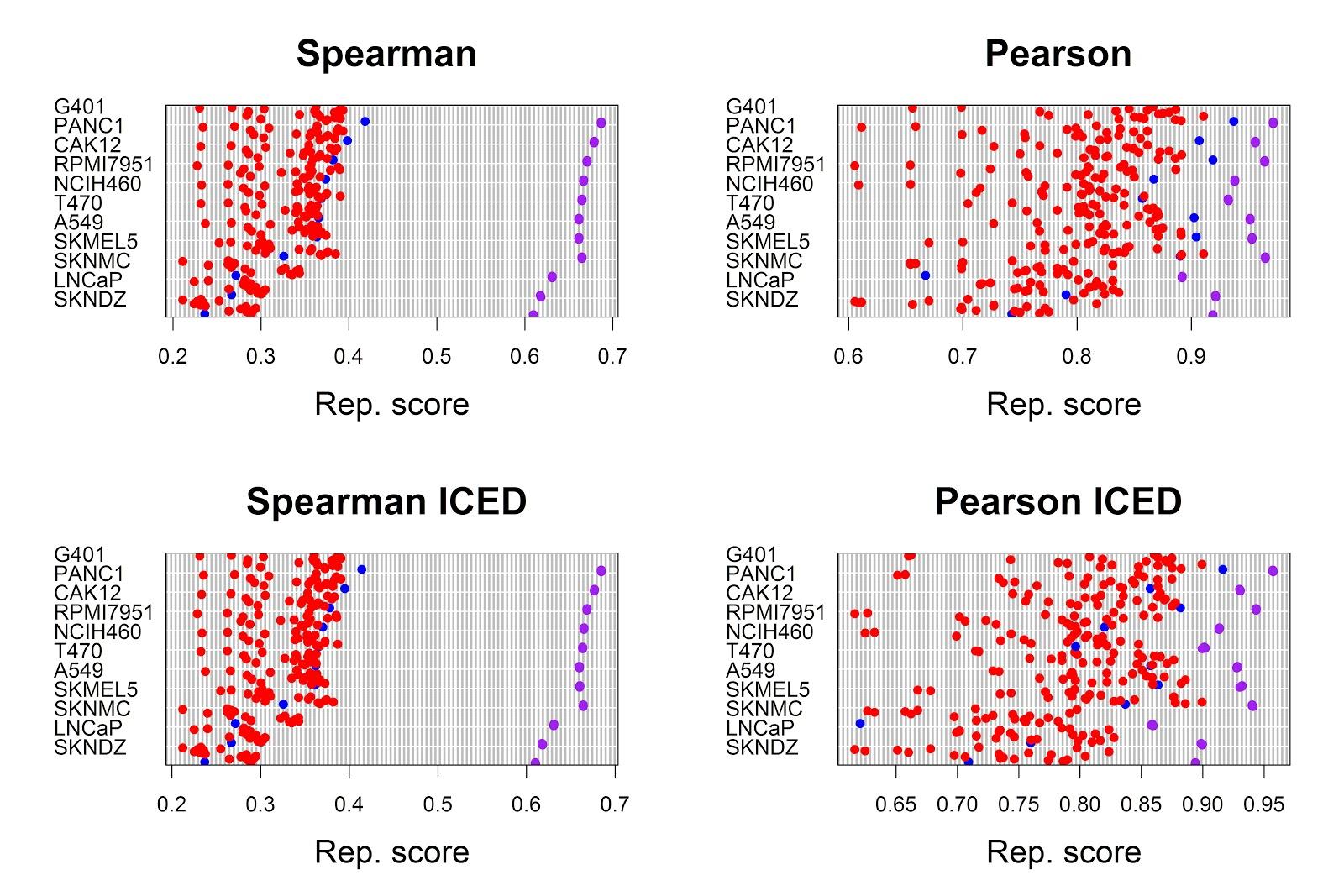


Figure S3**.** Comparison of Pearson and Spearman correlation coefficients on raw and normalized Hi-C data**.** Correlation coefficients assigned to biological replicate (blue), non-replicate (red) and pseudo-replicate (purple) pairs are plotted for each cell type. All of these experiments used HindIII digestion.


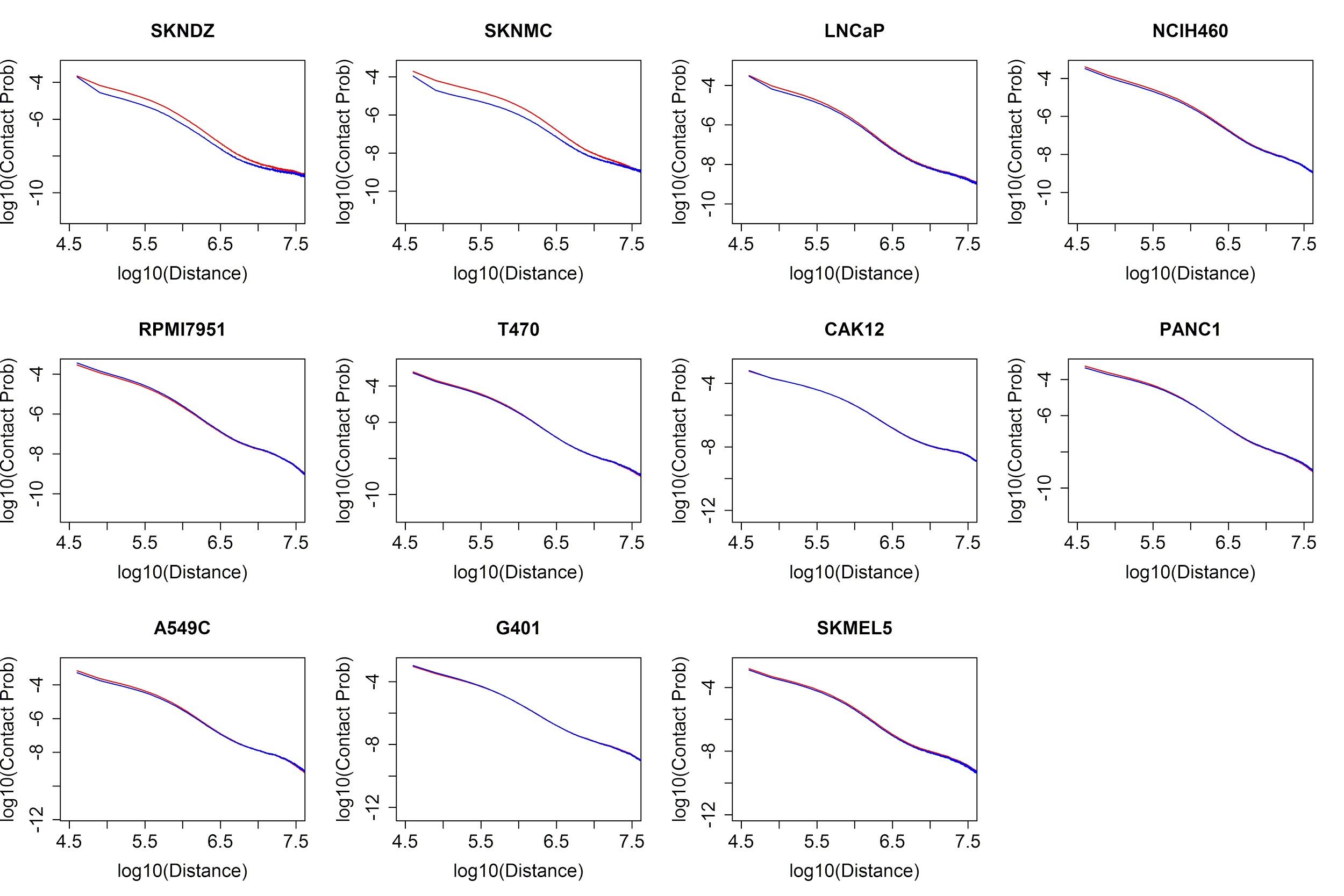


Figure S4. Contact probability curves for biological replicates pairs from each replicate pair. The curve for first replicate is shown in red and the second replicate is in blue.


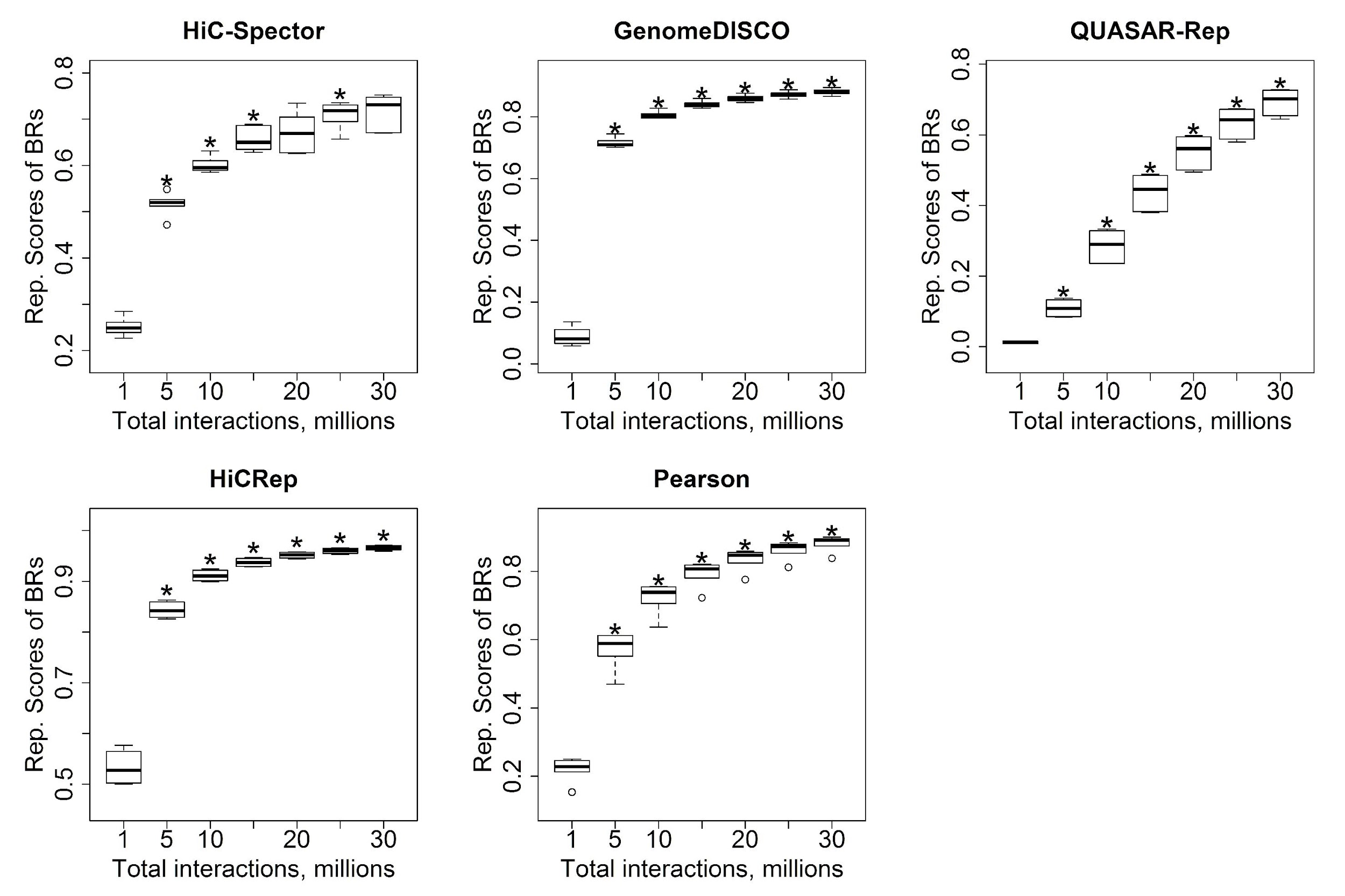


Figure S5. Boxplots showing the distribution of reproducibility scores assigned to six downsampled biological replicates at each coverage level. Asterisks above each distribution indicate that the distribution of reproducibility scores assigned to that distribution is significantly larger than the previous distribution according to a one-sided Wilcoxon signed rank test (P < 0.05)


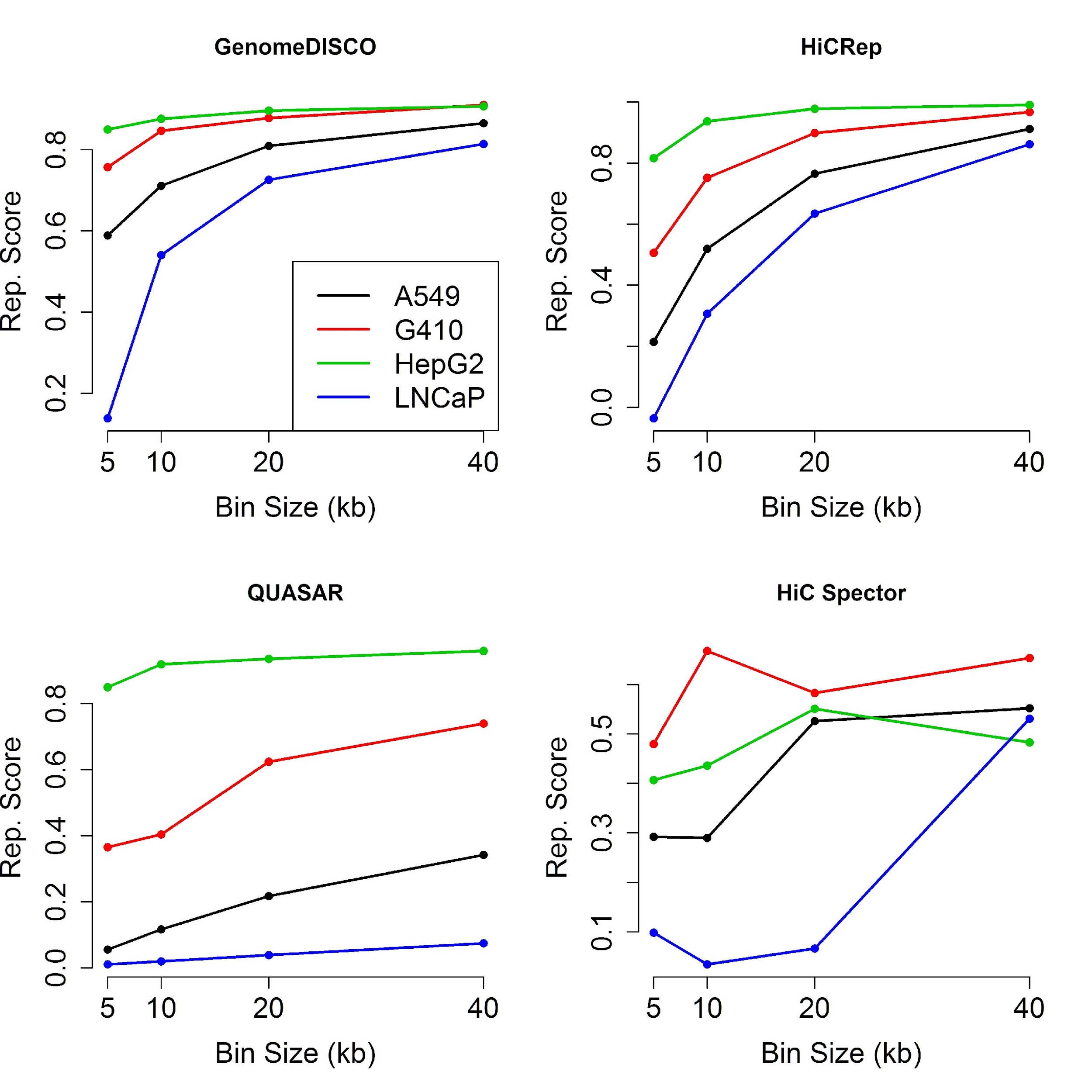


Figure S6. Relationship between reproducibility score and resolutionbin size. Each panel plots a specific reproducibility score as a function of resolution (5, 10, 20, 40 kb). Each of the four series corresponds to a biological replicate from a specified cell line.


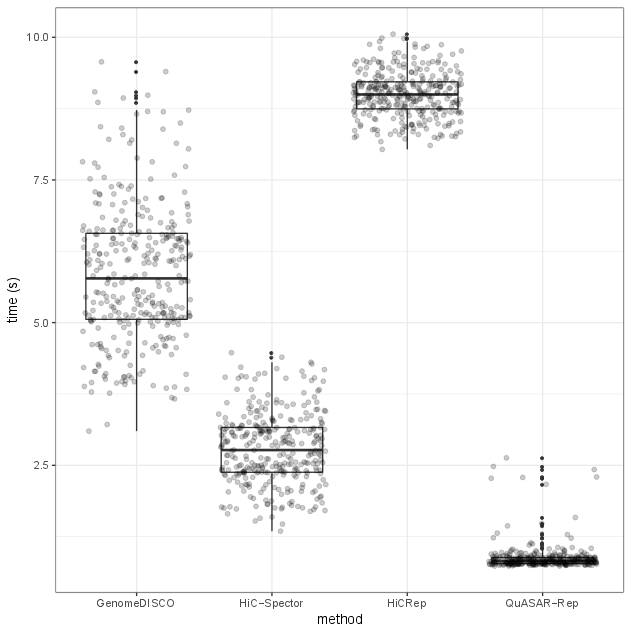


Figure S7. Run times for each reproducibility measure. Each boxplot shows the distribution of run times on 326 replicate pairs for each reproducibility measure. The times we report are wall-clock times, as reported by the “time” command in Unix. All tests were run on an Intel Xeon CPU E5-2683 v3 running at 2.00GHz.


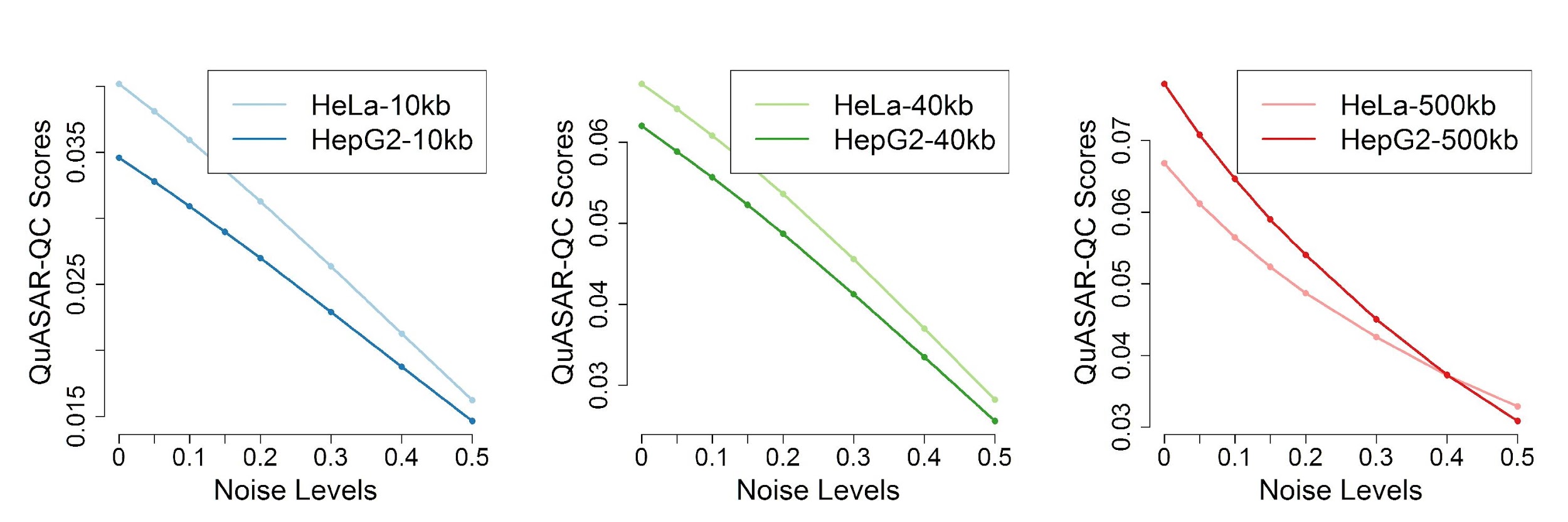
Figure S8. QuASAR-QC scores assigned to noise injected simulated datasets from deeply sequenced replicates. QuASAR-QC scores decrease with increasing levels of noise at all resolutions.


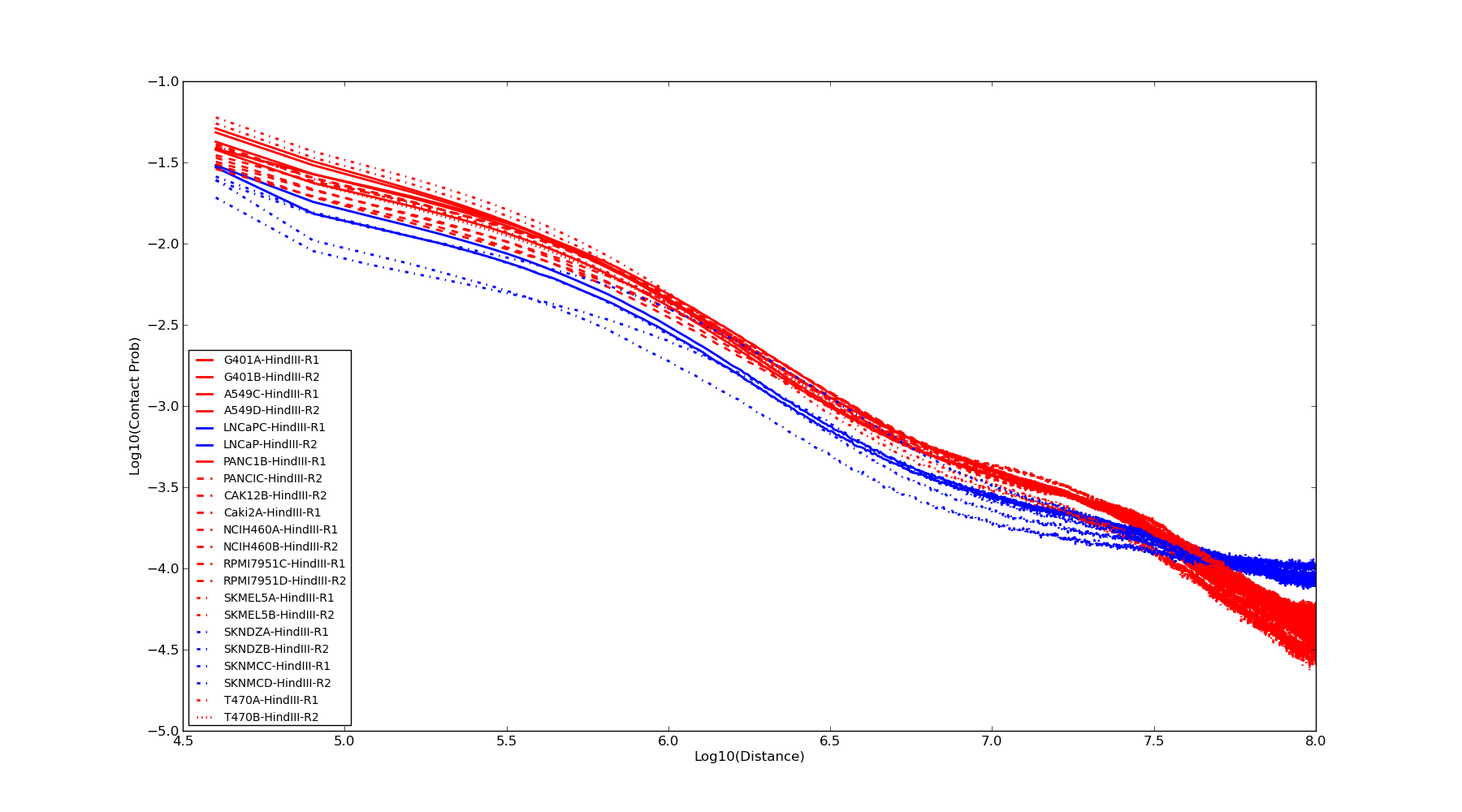


Figure S9. Curves showing the probability of contact between two loci against genomic distance. The blue curves are generated using Hi-C experiments done on SKNDZ, SKNMC and LNCaP cell lines.


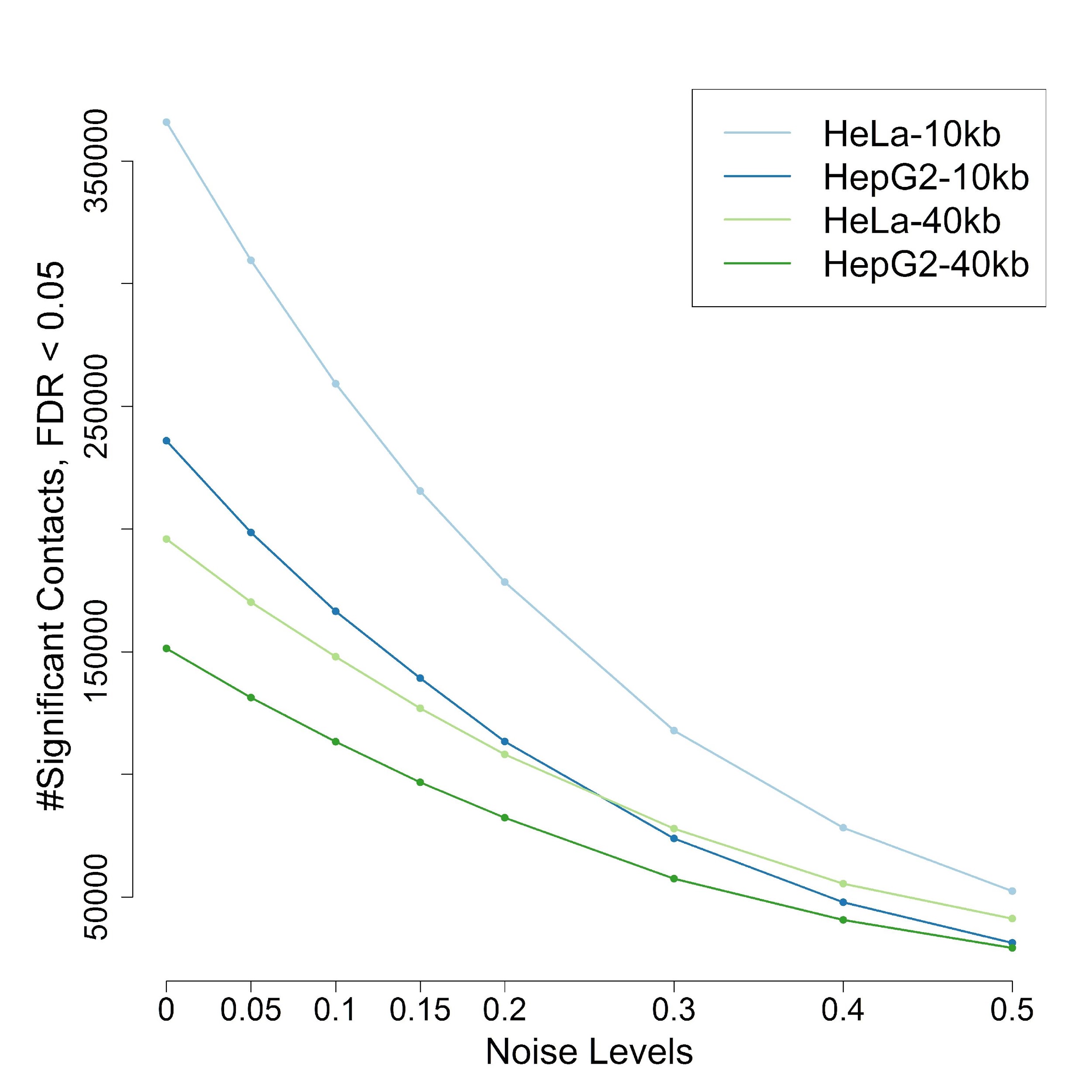


Figure S10. Total number of significant mid-range contacts identified by Fit-Hi-C with an FDR threshold of 0.05 from simulated datasets at 10kb and 40kb resolutions, generated from deeply sequenced datasets. The total number of contacts for each cell type and resolution decreases monotonically with increasing levels of noise.


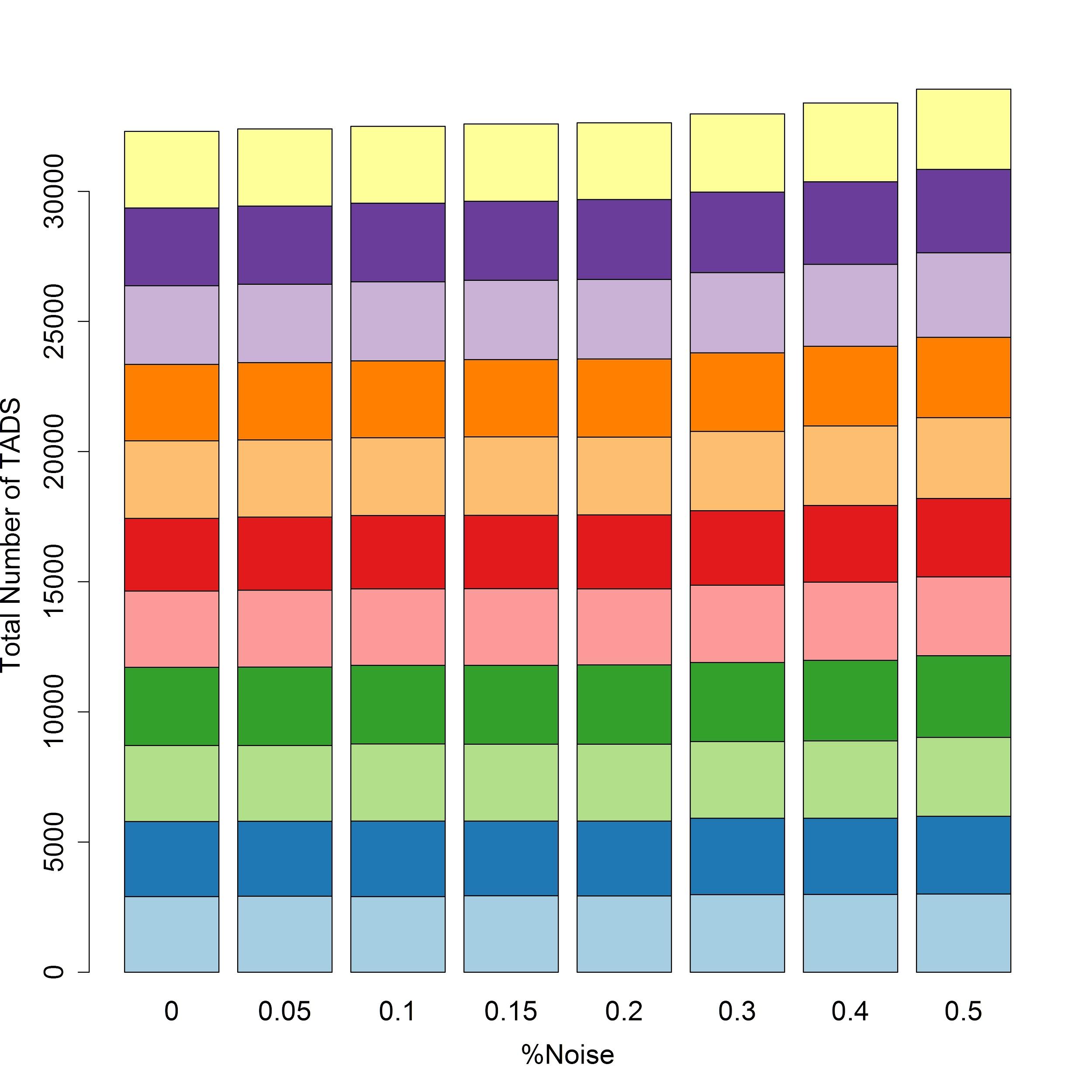


Figure S11. Bar plots showing the number of TADs for each cell line (coded by color) at each noise injection level.


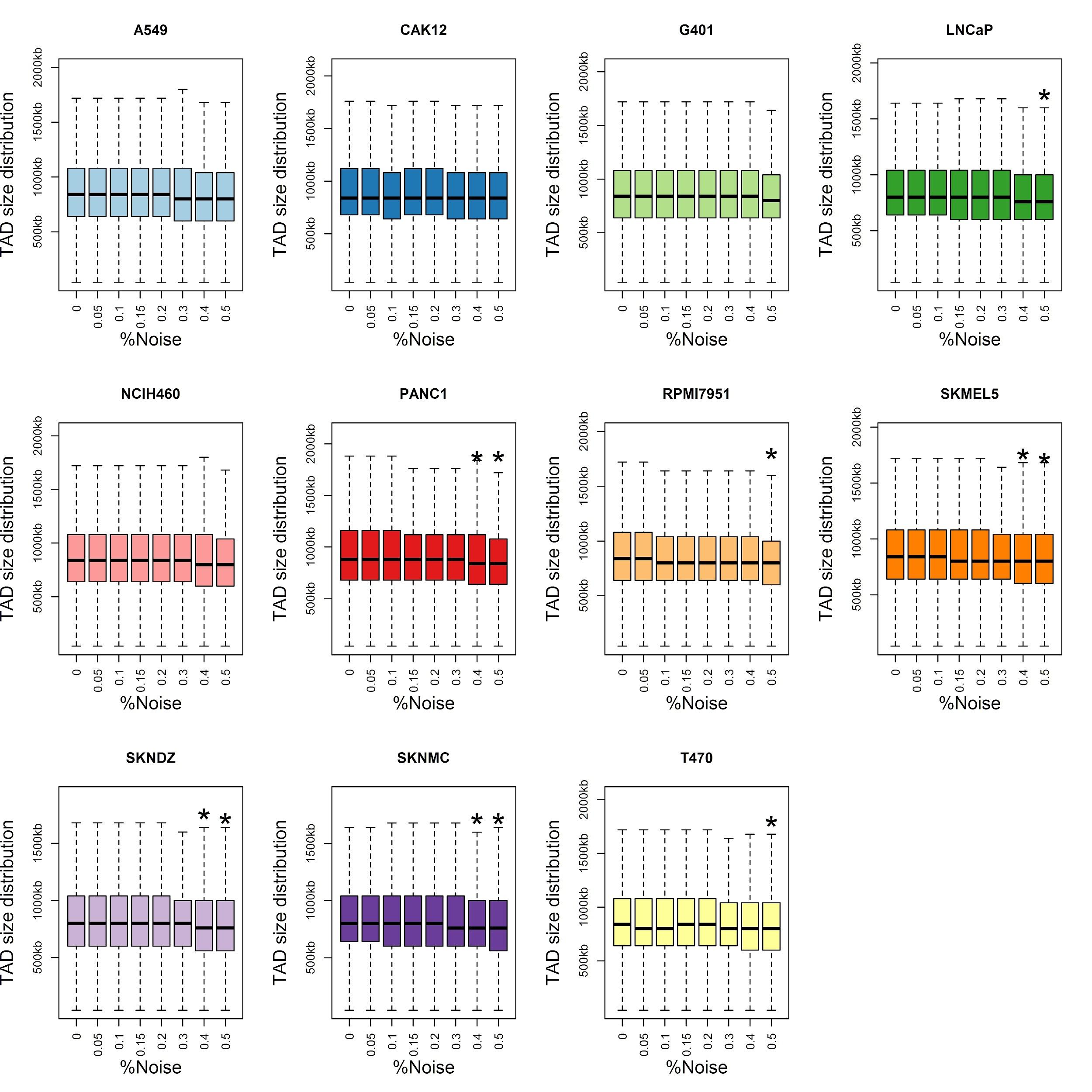


Figure S12. Boxplots showing the distribution of TAD sizes at each noise injection level. Each plot corresponds to a simulated dataset from an individual cell type. Distributions marked with an asterisk are significantly different from the original distribution TAD sizes detected from the noise-free replicate (KS test, P < 0.01).


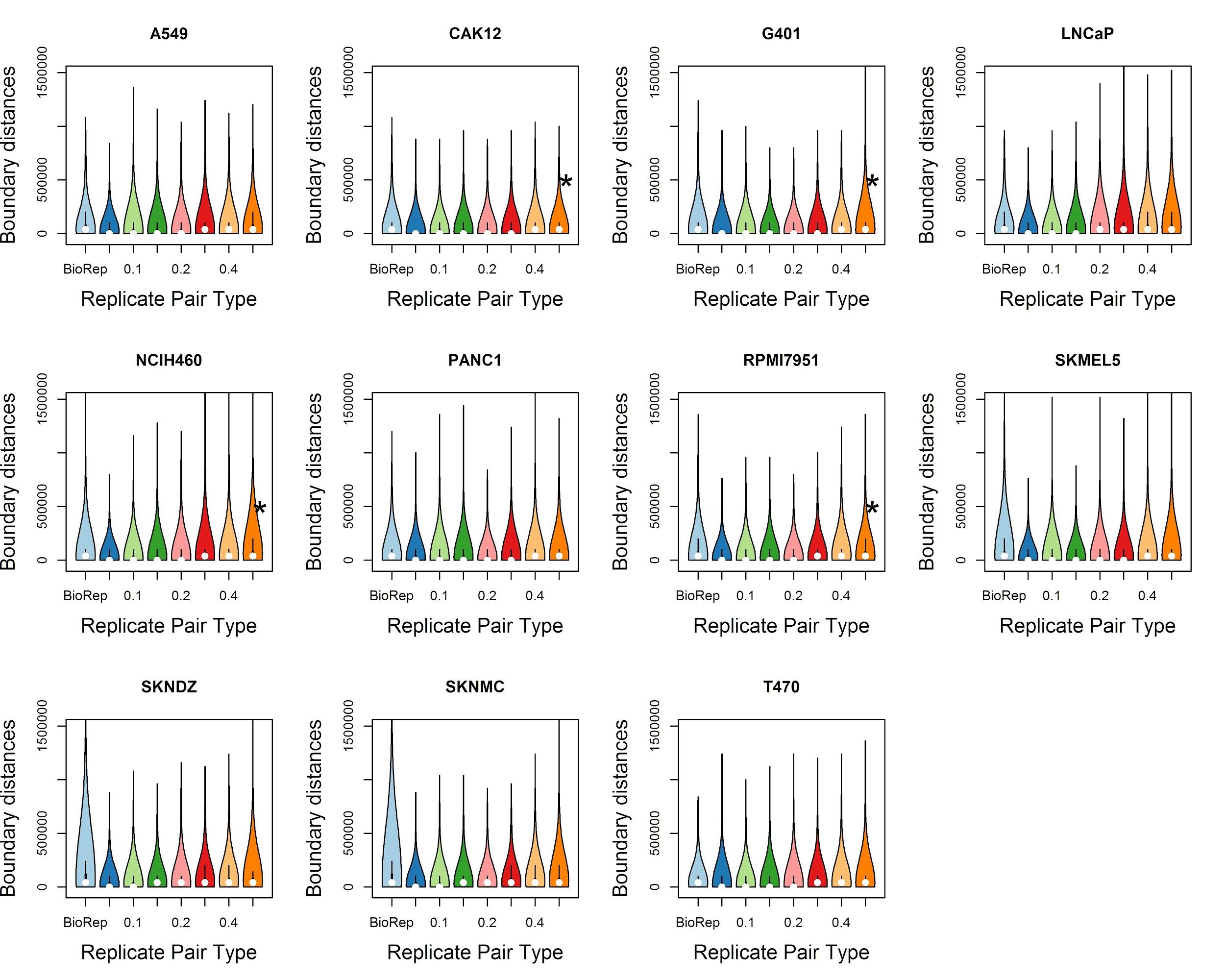


Figure S13. Violin plots showing the distribution of distances between domain boundaries between biological replicates and simulated replicates. Each panel corresponds to a single cell type.


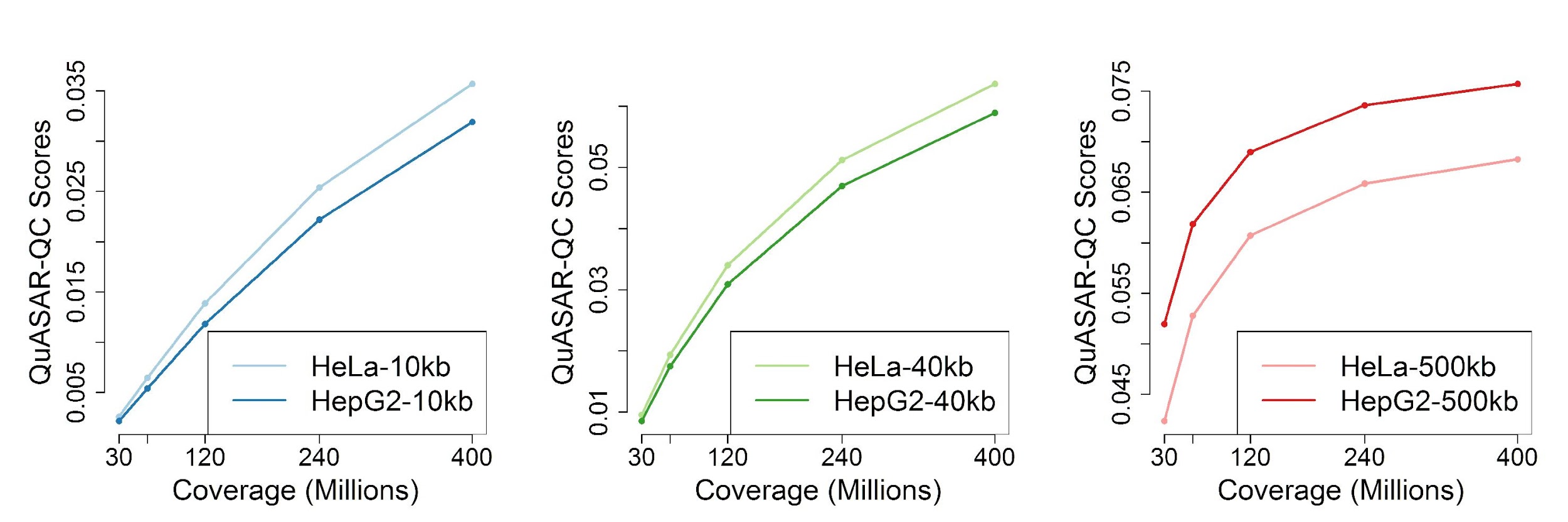


Figure S14. Curves showing the QuASAR scores assigned to deeply sequenced cell types downsampled to 30, 60, 120, 240 and 400 million interactions at 10kb, 40kb and 500kb resolutions.


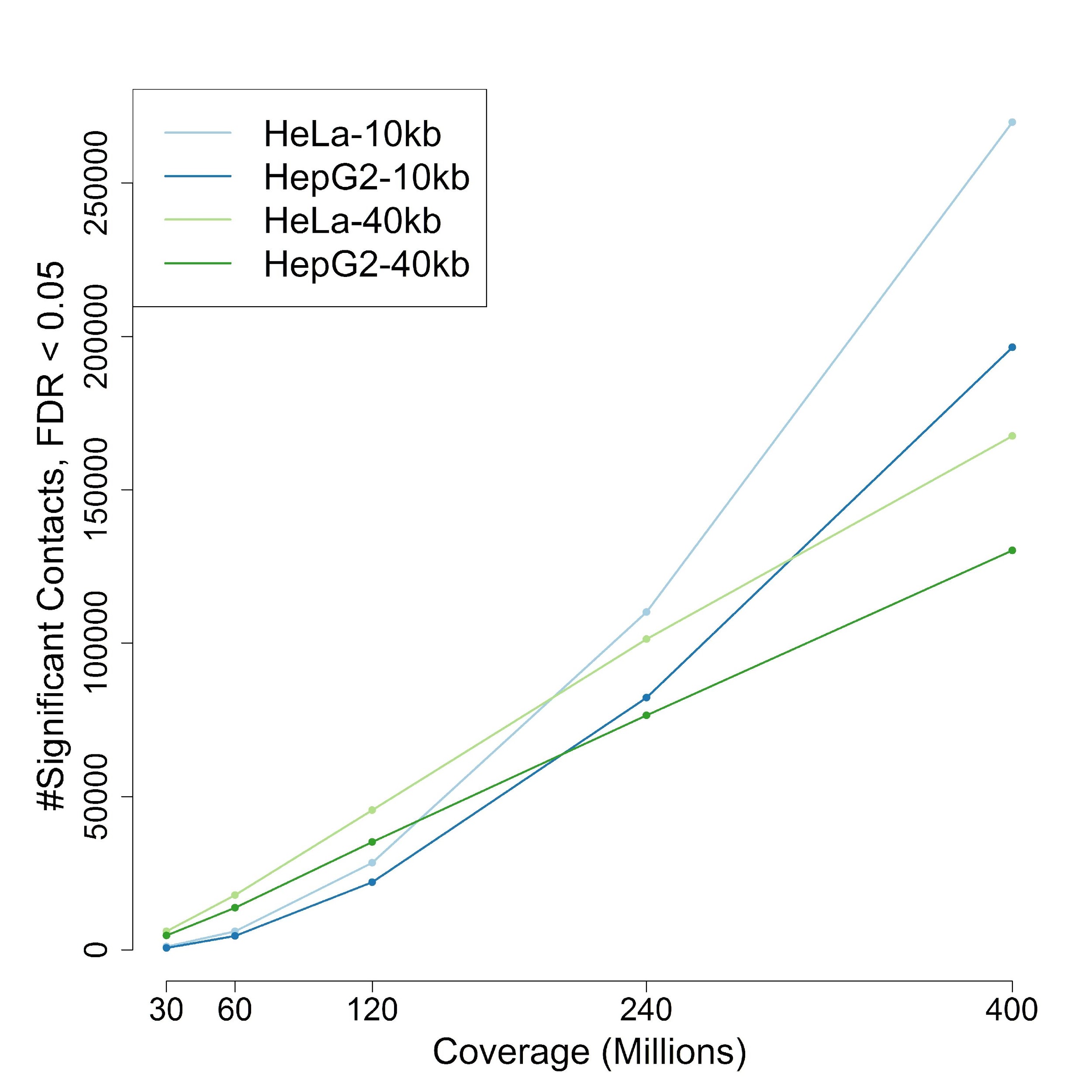


Figure S15. Curves showing the total number significant mid-range interactions detected by FIt-Hi-C from deeply sequenced cell types downsampled to 30, 60, 120, 240 and 400 million interactions at 10kb and 40kb resolutions.


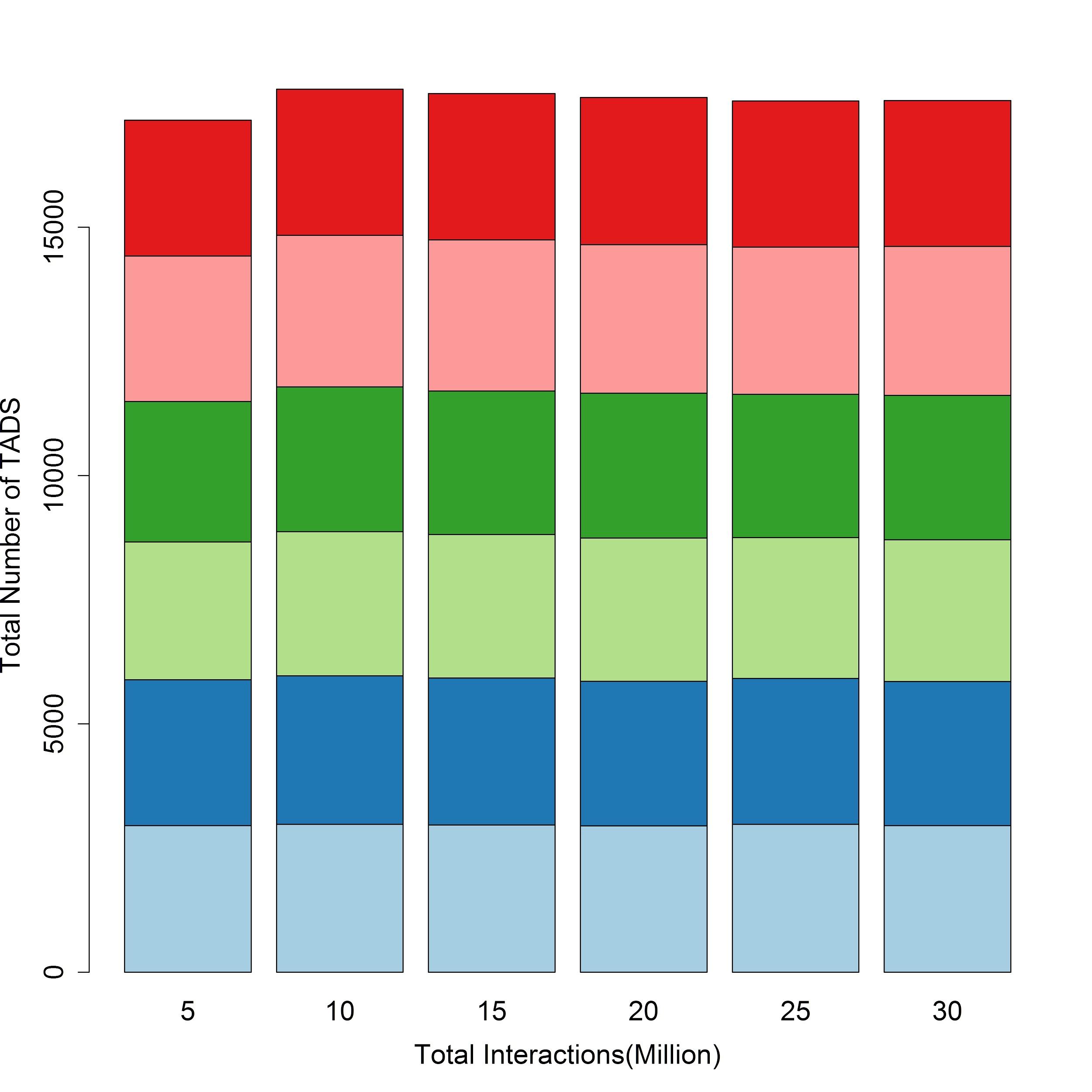


Figure S16. Barplots showing the number of TADs for each downsampled cell line (coded by color) at each coverage level.


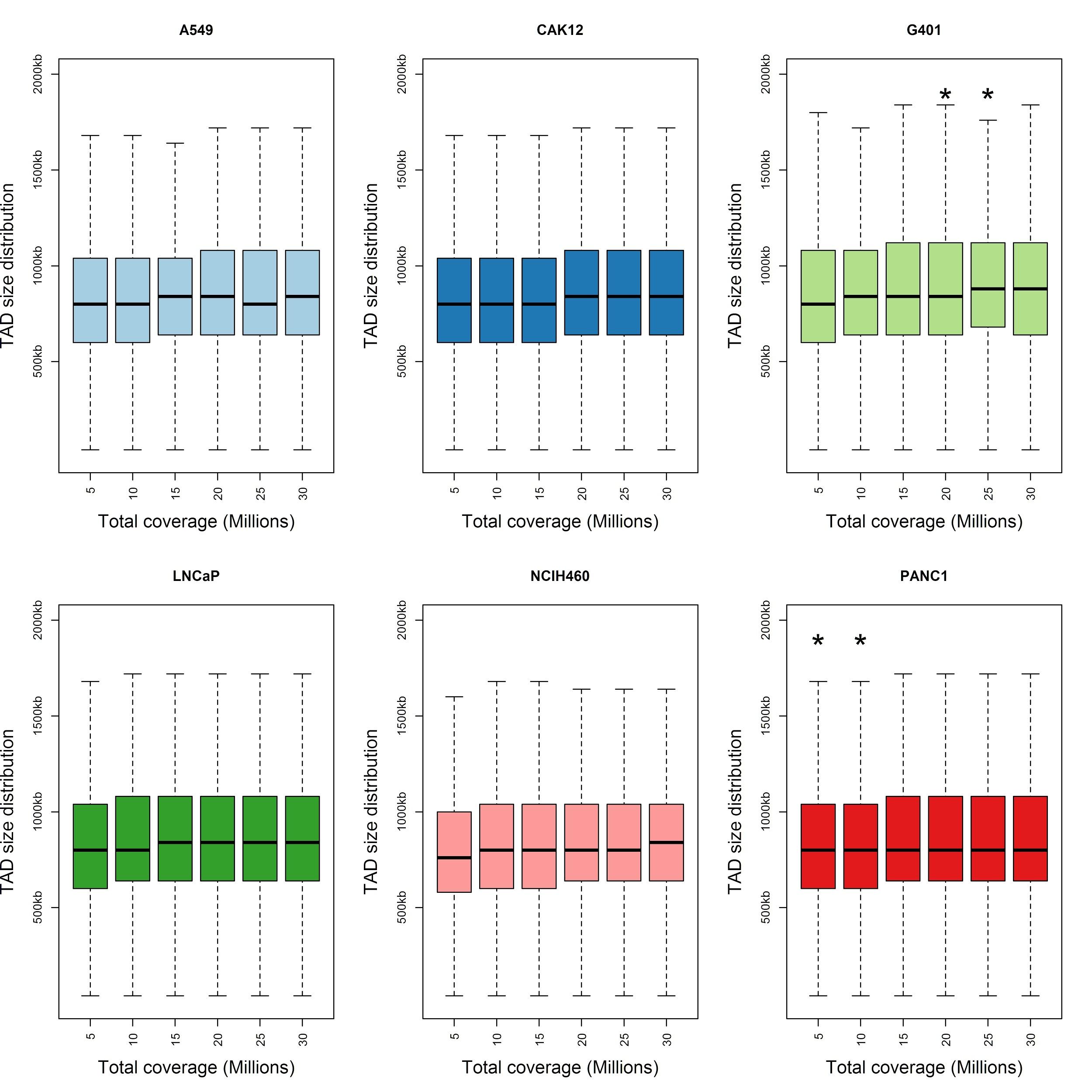


Figure S17. Boxplots showing the distribution of TAD sizes at downsampling level. Each plot corresponds to a simulated dataset from an individual cell type.


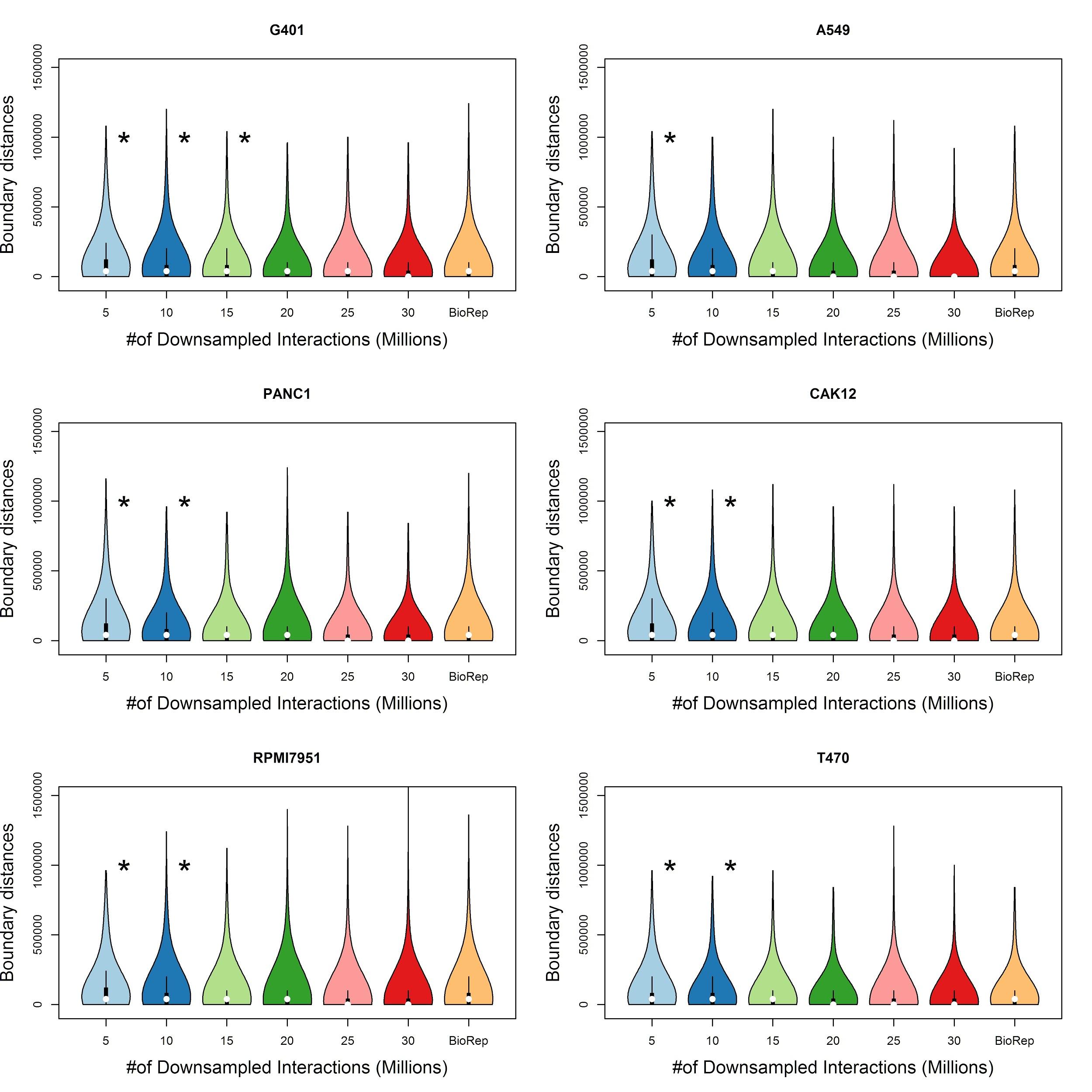


Figure S18. Violin plots showing the distribution of distances between domain boundaries between biological replicates and downsampled replicates. Each panel corresponds to a single cell type.

**
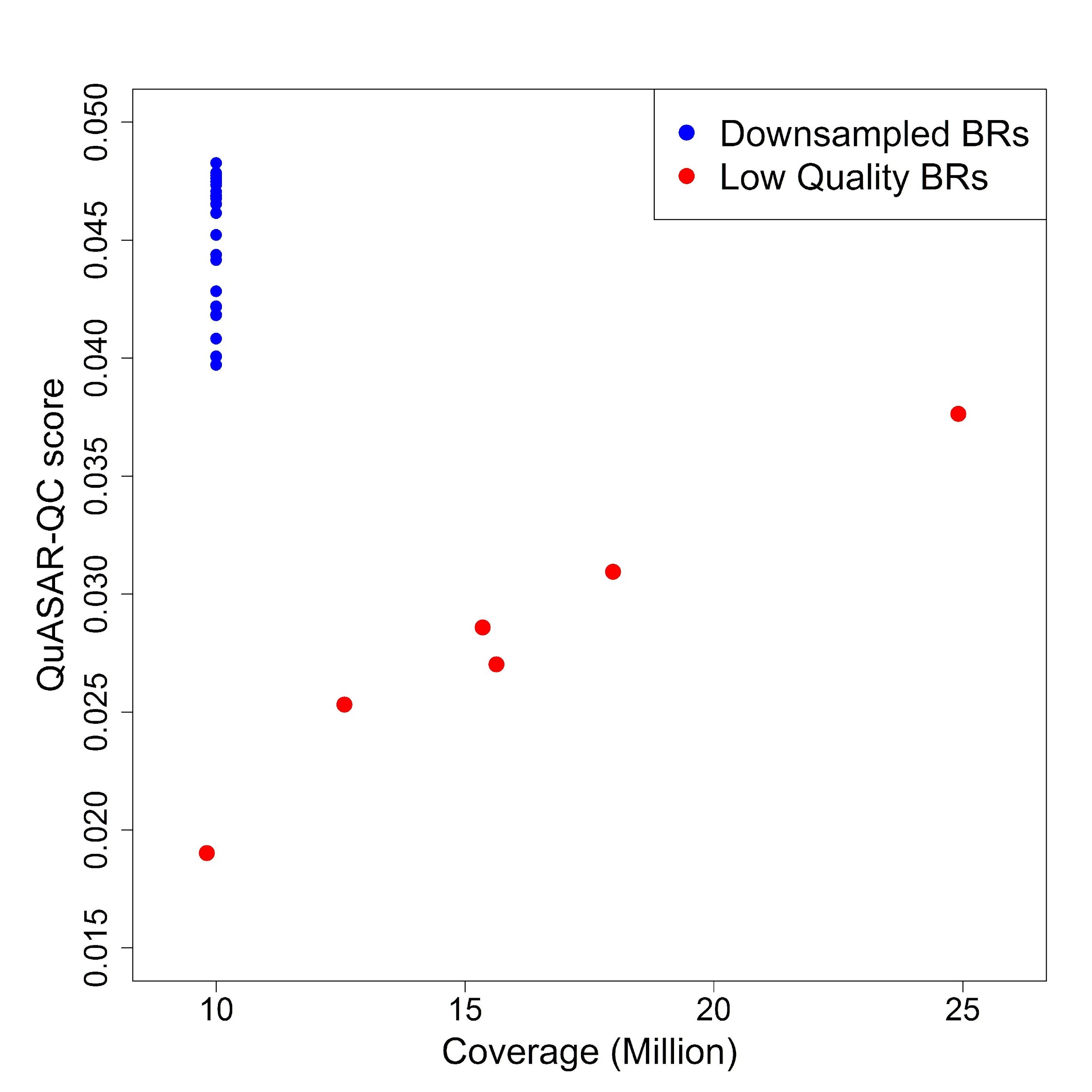
**

Figure S19**.** “Lower quality” data sets exhibit low QuASAR-QC scores compared to scores from downsampled data**.** The figure plots the QuASAR-QC score as a function of coverage. Red points are biological replicates from three cell lines designated as “low quality” Hi-C data. Blue points are from other cell types, after downsampling to 10 million interactions. Despite having higher coverage, lower quality datasets have lower QuASAR-QC scores.

| Biosample | Tissue/Morphology | 1^st^ replicate coverage | 2nd replicate coverage | ENCODE sample IDs | Resolutions |
| --- | --- | --- | --- | --- | --- |
| A549 | Lung/Epithelial | 33,028,385 | 30,167,658 | ENCSR444WCZ | 40kb |
| CAKI2 | Kidney/Epithelial | 36,297,274 | 47,032,721 | ENCSR401TBQ | 40kb |
| G401 | Kidney/Epithelial | 61,278,507 | 52,988,386 | ENCSR079VIJ | 40kb |
| LNCaP | Prostate/Epithelial | 17,976,198 | 15,357,134 | ENCSR346DCU | 40kb |
| NCIH460 | Lung/Epithelial | 41,579,896 | 28,892,164 | ENCSR489OCU | 40kb |
| PANC1 | Pancreas/Epithelial | 37,454,217 | 50,535,714 | ENCSR440CTR | 40kb |
| RPMI7951 | Skin/Epithelial | 31,953,729 | 48,764,886 | ENCSR862OG | 40kb |
| SKMEL5 | Skin/Stellate | 45,742,471 | 10,651,488 | ENCSR312KHQ | 40kb |
| SKNDZ | Brain/Epithelial | 15,631,291 | 9,813,185 | ENCSR105KFX | 40kb |
| SKNMC | Brain/Epithelial | 24,914,561 | 12,578,436 | ENCSR834DXR | 40kb |
| T47D | Mammary Gland/Epithelial | 33,902,719 | 35,957,065 | ENCSR549MGQ | 40kb |
| HepG2 | Liver/Epithelial | 412,741,167 | 456,705,426 | ENCSR194SRI | 10kb,40kb,500kb |
| HeLa | Cervix/Epithelial | 515,837,715 | 494,774,796 | ENCSR693GXU | 10kb,40kb,500kb |

Table S1. Thirteen human cancer cell types that Hi-C experiments were performed on, together with the tissue type and lineage the cells were immortalized from. Two replicate experiments were performed in each cell type. The coverage columns list the total number of intra-chromosomal interactions for the 1^st^ and the 2^nd^ replicate for each cell type. ENCODE sample ID of each experiment is provided in the corresponding column. The first 11 cell types with lower coverage vaues are binned at only 40kb resolution, whereas the last 2 cell types with large number of Hi-C interactions are binned at three different resolutions.

|  | 30 ✕ 10^6^ | 25 ✕ 10^6^ | 20 ✕ 10^6^ | 15 ✕ 10^6^ | 10 ✕ 10^6^ | 5 ✕ 10^6^ |
| --- | --- | --- | --- | --- | --- | --- |
| HiC-Spector | 0.472 | 0.472 | 0.452 | 0.446 | 0.43 | 0.371 |
| GenomeDISCO | 0.833 | 0.826 | 0.815 | 0.798 | 0.767 | 0.679 |
| QuASAR-Rep | 0.63 | 0.573 | 0.497 | 0.392 | 0.253 | 0.096 |
| HiCRep | 0.882 | 0.877 | 0.868 | 0.855 | 0.829 | 0.765 |

Table S2. Empirical thresholds for distinguishing non-replicates from biological replicates for each measure at a given coverage level. Each column corresponds to empirical threshold inferred by using biological replicates and non-replicates that have been downsampled to the value in the column header (see Methods).
